# Dynamic Compensation of a Piezoelectric Accelerometer Obtained through a General Probabilistic Approach †

**DOI:** 10.3390/s23083950

**Published:** 2023-04-13

**Authors:** Francesco Crenna, Giovanni Battista Rossi, Marta Berardengo

**Affiliations:** DIME—Department of Mechanical, Energy, Management and Transportation Engineering, University of Genova, 16145 Genova, Italy; g.b.rossi@unige.it (G.B.R.); marta.berardengo@unige.it (M.B.)

**Keywords:** dynamic measurement, measurement modeling, probabilistic models, dynamic compensation, accelerometers

## Abstract

Dynamic compensation is the (partial) correction of the measurement signals for the effects due to bandwidth limitations of measurement systems and constitutes a research topic in dynamic measurement. The dynamic compensation of an accelerometer is here considered, as obtained by a method that directly comes from a general probabilistic model of the measurement process. Although the application of the method is simple, the analytical development of the corresponding compensation filter is quite complex and had been previously developed only for first-order systems, whilst here a second-order system is considered, thus moving from a scalar to a vector problem. The effectiveness of the method has been tested both through simulation and by a dedicated experiment. Both tests have shown the capability of the method of significantly improve the performance of the measurement system when dynamic effects are more prevalent than additive observation noise.

## 1. Introduction

Dynamic measurement can be defined as measurement where the measurand value varies during the observation time and the investigation of such variations is the main goal of measurement, whilst in static measurement, the measurand is assumed to remain constant and assessing its value is the major goal. Dynamic measurement is the object of study nowadays, due to the importance of its application and to the scientific and technological challenges it still poses. A metrological approach, where issues such as dynamics calibration, uncertainty reduction, and evaluation are particularly considered, can contribute to improve greatly the quality of such measurements.

Papers in this area include, e.g., a report of the results of a European-funded project on this subject [1], careful consideration of calibration issues [2], and general modelling aspects [3]. The adoption of models from generic system theory has been suggested [4], and related terminological issues have been considered [5].

A special problem in dynamical measurement is the (partial) correction of the measurement signals for the effects due to bandwidth limitations of measurement systems and the accuracy of this correction. This is called dynamic compensation and it can be obtained by dedicated analog devices, called (dynamic) compensators [6], or through digital signal processing [7]. In [8], it was stressed that the approach to be followed may depend upon the form of the available information, either frequency response, differential, or finite-difference equations. In [9], a finite impulse response (FIR) filter was developed based on an optimization process. Special application cases were also considered, including hydrophone measurements [10], contact probes [11], force sensors [12], and pressure sensors [13].

Yet, most of the current literature on this subject seems to take the approach, explicitly declared in [4], that, whilst looking forward to developing “a self-evident definition of… measurement *plus* observation”, it appears that “dynamic systems and subsystems can model and visualize this structurally demanding result within Signal and System Theory best”. In other words, what is needed is simply a proper application of methods from system and signal theory. In this paper, instead, a somewhat different approach is pursued as a step in a series of studies that the authors have carried out in the last, say, ten years, with the aim of developing a unified theory/model of measurement, where static and dynamic measurements share the same structure. In this regard, a general probabilistic structure of the measurement process was proposed in [14,15], and, more recently, a logistic interpretation of probability was proposed in [16,17]. Then, static and dynamic measurements were investigated separately, yet maintaining the same basic framework for both of them. Investigation on static measurement included, e.g., a special but important application area: that of instruments subject to the Measuring Instrument Directive (MID) [18], for which guidelines were proposed [19], and a proposal for a revision of the GUM [20] in order to make it more user oriented [21]. Concerning dynamic measurement, the application of the approach proposed by the authors requires the selection of a general model. In this regard, a Markovian model with additive observation noise was proposed. The development is not trivial, although the final result comes out being manageable and even simple. A complete development was proposed for first order models [22]. The purpose of this paper is to extend the approach to second-order systems. This step is important, since it implies a transition from scalar to vector systems and because a second order approximation nearby the first resonance frequency can be in some cases appropriate. The results to be presented here were partially anticipated in a conference communication [23], where the approach was validated by simulation only, whilst an experimental validation, concerning an important class of measuring systems, namely accelerometers, is here included. Such an experimental validation seems particularly important when the focus is on measurement, as it is the case here.

Therefore, the paper is organized as follows. In Section 2, the proposed method is presented. In Section 3, validation by simulation is performed by considering two different test cases. In Section 4, the experimental validation is carried out on piezoelectric accelerometers by examining three test cases. Lastly, in Section 5, conclusions are drawn.

## 2. The Proposed Method

### 2.1. The Generic Modelling Framework

It is suggested that measurement can be parsed in two phases, called *observation* and *restitution*. In the observation phase the “object” carrying the property to be measured, x, interacts with the measurement system in such a way that an observable output, the instrument indication, y, is produced based on which a measurement value can be assigned to the measurand. In probabilistic terms, observation can be described by the conditional probability density $p\left(y|x\right)$. The successive phase, where the result is produced based on the instrument indication and accounting for calibration results (calibration curve), is here called restitution and described by the conditional probability density $p\left(x|y\right)$. Restitution can thus be performed through the Bayes–Laplace rule, assuming a uniform, uninformative prior for the measurand, as is natural in measurement, obtaining [15,24]:

(1) $$p\left(x|y\right)=\frac{p\left(y|x\right)}{\int_{X}^{}p\left(y|x\right)dx}\;,$$

where X denotes the domain of x. Interestingly enough, this same framework remains valid when considering different types of measurements, although at different levels of complexity. In practice, scalars may become vectors or matrices, and the model will be more complex, but the basic structure remains the same, which allows us to treat different types of measurements in a consistent way. For example [14],

- for static measurement, based on a single observation, x and y are both scalar quantities;
- for static measurement, based on N repeated observation, x remains a scalar quantity, but y becomes a vector, y, with N components;
- for direct dynamic measurement, where the goal is to measure the time history of some quantity, based on N time-sampled observations, both x and y become vectors with N components;
- for indirect dynamic measurement, where, for example, the final goal is to obtain a spectrum of the phenomenon, with, say, M spectral lines, both x and y are still vectors, but x, which is now the spectrum, has now M components.

Here we are interested in direct dynamic measurement.

### 2.2. The Proposed Method

Let then x be a quantity that characterizes a dynamic phenomenon and $x_{i}$ the value it takes at instant $t_{i}=i∆t$, where i is an integer, Δt the sampling interval, and $y_{i}$ the corresponding indication obtained by a measurement system suited for that quantity. Let x and y denote the vectors that collect the measurand values and the instrument indications respectively, acquired in a time interval of duration T=N∆t.

Then, the generic structure presented in (1) now becomes:

(2) $$p\left(x|y\right)=\frac{p\left(y|x\right)}{\int_{X}^{}p\left(y|x\right)dx}.$$

To implement this model, it is necessary to calculate the distribution $p\left(y|x\right)$. Let us introduce the following convenient notation:

(3) $$\begin{array}{c} x^{i}=\left(x_{1},x_{2},\ldots ,x_{i}\right),\\ y^{i}=\left(y_{1},y_{2}\ldots ,y_{i}\right). \end{array}$$

As a general property of joint probability distributions, the following factorization holds true [16]

(4) $$p\left(y|x\right)=p\left(y_{1}|x\right)\ldots p\left(y_{i}|y^{i-1},x\right)\ldots p\left(y_{N}|y^{N-1},x\right).$$

With the additional assumption of causality, i.e., that the indication y at instant $t_{i}$ depends on the measurand values only up to instant $t_{i}$, (4) further simplifies into

(5) $$p\left(y|x\right)=p\left(y_{1}|x_{1}\right)\ldots p\left(y_{i}|y^{i-1},x^{i-1}\right)\ldots p\left(y_{N}|y^{N-1},x^{N-1}\right).$$

In [16] the problem was solved in the case of first-order systems. Here a second-order system expressed by a second-order difference equation of the type

(6) $$z_{i}=a_{1}z_{i-1}+a_{2}z_{i-2}+b_{1}x_{i-1}+b_{2}x_{i-2}\;$$

is considered, where z is some state variable of the device. In the case of accelerometers, of direct interest in this study, z may be the displacement of the seismic mass within the sensor case or the force transmitted to a secondary piezoelectric transducer, depending upon the technology. Let the indication y be linked to z by the observation equation:

(7) $$y_{i}=kz_{i}+v_{i},{\;}_{}\;$$

where k is the sensitivity and $v_{i}$ is a sequence of random variations which are uncorrelated realization of a zero-mean probabilistic variable, with variance $\sigma^{2}$.

Then, by combining (6) and (7), the input–output equation results in

(8) $$y_{i}=a_{1}y_{i-1}+a_{2}y_{i-2}+kb_{1}x_{i-1}+kb_{2}x_{i-2}+w_{i},$$

where

(9) $$w_{i}=v_{i}-a_{1}v_{i-1}-a_{2}v_{i-2}.$$

Therefore, the *i*-th term of (5), for i>2, is

(10) $$p\left(y_{i}|y^{i-1},x^{i-1}\right)=p_{w}\left(y_{i}-a_{1}y_{i-1}-a_{2}y_{i-2}-kb_{1}x_{i-1}-kb_{2}x_{i-2}\right)=p\left(y_{i}|y_{i-1},y_{i-2},x_{i-1},x_{i-2}\right).$$

If we include (10) in (5) and in (2), we obtain

(11) $$p\left(x|y\right)\propto \prod_{i=3}^{N}p_{w}\left(y_{i}-a_{1}y_{i-1}-a_{2}y_{i-2}+kb_{1}x_{i-1}+kb_{2}x_{i-2}\right)\;.$$

To extract the marginal distribution for the generic term $x_{i}$, let us re-write (8) one step ahead:

(12) $$y_{i+1}=a_{1}y_{i}+a_{2}y_{i-1}+kb_{1}x_{i}+kb_{2}x_{i-1}+w_{i+1}$$

and solving it for $x_{i}$, we obtain

(13) $$x_{i}={\left(kb_{1}\right)}^{-1}\left(y_{i+1}-a_{1}y_{i}-a_{2}y_{i-1}\right)+b_{1}^{-1}b_{2}x_{i-1}+{\left(kb_{1}\right)}^{-1}w_{i+1}.$$

Using now the same approximation suggested in [16], i.e., substituting ${\hat{x}}_{i-1\;}$ to $x_{i-1}$, we obtain the following recursive estimator for $x_{i}$:

(14) $${\hat{x}}_{i}={\left(kb_{1}\right)}^{-1}\left(y_{i+1}-a_{1}y_{i}-a_{2}y_{i-1}\right)+\;b_{1}^{-1}b_{2}{\hat{x}}_{i-1},$$

which constitutes a non-causal second order linear filter. Checking the validity of this approximation will be one of the major goals of simulation.

If ${\hat{x}}_{i}$ is substituted for $x_{i}$ in (10), after assuming a Gaussian distribution for v and consequently for w, we lastly obtain

(15) $$p\left(x_{i}|y\right)=\varsigma^{-1}\varphi \left(\varsigma^{-1}\left(x_{i}-{\hat{x}}_{i}\right)\right),$$

where [16]

(16) $$\sigma_{\varsigma }^{2}\leq \frac{1+a_{1}^{2}+a_{2}^{2}}{k^{2}b_{1}{}^{2}}\sigma^{2}$$

and ${\hat{x}}_{i}$ is given by (14).

### 2.3. Overall View of the Method

**Figure 1:**
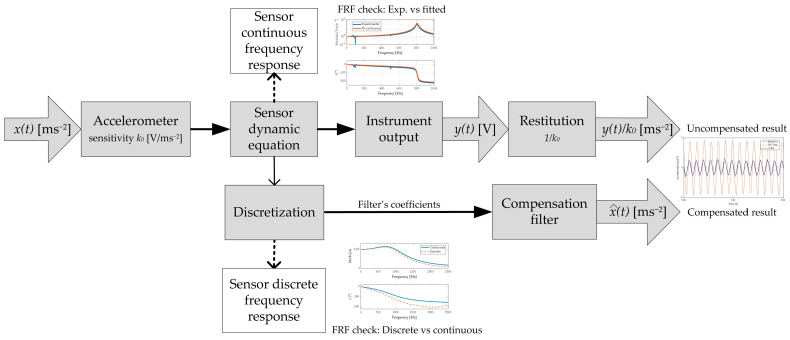
Overall view of the method.

An overall view of the method is presented in Figure 1. The measuring device, operating in continuous time, is modelled through its differential dynamic equation and its corresponding continuous-time frequency response. This equation is discretized by a suited method, and the corresponding finite-difference equation is used to develop a filter that performs the required dynamic compensation.

Such compensation is a part of the restitution process that yields the final result. A discrete-time frequency response corresponds to the finite difference equation, and its comparison with the continuous time response is an important checking step for the validation of the discretization method.

The effectiveness of the method will be checked in the next sections, both by simulation and by a dedicated experiment. In the test cases, different kind of input signals will be considered and the method will be validated in all the cases.

## 3. Testing the Method by Simulation

### 3.1. Design of the Simulation Tests

The method has been tested through simulation first and then experimentally validated, considering the case of a piezoelectric accelerometer, a typical important instrument for mechanical measurements, which has been already considered as an interesting example of dynamic measurements [25].

The dynamic (mechanical) behaviour of the sensor can be described, in continuous time, by the second order differential equation [6]

(17) $$\ddot{F}\left(t\right)=-2\zeta \left(2\pi f_{0}\right)\dot{F}\left(t\right)-{\left(2\pi f_{0}\right)}^{2}F\left(t\right)+m{\left(2\pi f_{0}\right)}^{2}a\left(t\right)\;,$$

where:

- $a\;(m/s^{2})$ is the acceleration to be measured,
- F (N) is the force detected by the piezoelectric transducer,
- m (kg) is the seismic mass of the inertial sensor,
- $f_{0}$ (Hz) is its natural frequency, and
- ζ is the damping factor.

The equation can be put in the standard form:

(18) $$\ddot{z}=\alpha_{1}\dot{z}+\alpha_{2}z+\beta x\;,$$

where dependence upon time is implied, x=a is the measurand and z=F is the state variable of the sensor relevant for the transduction, to be done through a secondary piezoelectric transducer, $\alpha_{1}=-2\zeta \left(2\pi f_{0}\right)$, $\alpha_{2}=-{\left(2\pi f_{0}\right)}^{2}$ and $\beta =+m{\left(2\pi f_{0}\right)}^{2}.$ For the simulation, the following values for the system’s parameters were assumed as: $f_{0}=1\;\mathrm{kHz}$, ζ=0.5, m=0.050 kg. The state variable of the sensor, z=F, is measured by the secondary piezoelectric transducer with its electronics, obtaining an overall sensitivity $k_{0}=0.01\;{\mathrm{Vs}}^{2}/m,\;$ which is a typical value for low–mid frequency devices. The system was tested in respect of both a sinusoidal and a step-like input.

### 3.2. Simulated Periodic Phenomenon

The measurand was modeled firstly as a periodic process,

(19) $$x\left(t\right)=x_{0}\sin2\pi f_{x}t\;,$$

with $x_{0}=1{\;\mathrm{ms}}^{-2}$, and $f_{x}=600\;\mathrm{Hz}.$ In particular, the testing frequency was assumed in the range where dynamical effects are relevant for checking the effectiveness of the method. For the noise, a rms value of 0.2 mV was assumed to check the influence of noise which is a relevant aspect of dynamic compensation.

**Figure 2:**
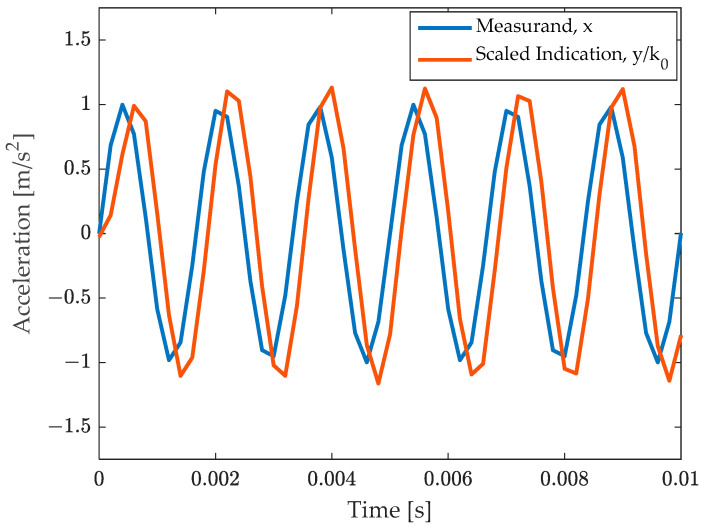
Comparison of the measurand with the (uncompensated) scaled instrument indication.

The system response was calculated by integrating the differential Equation (18) by a fourth order Runge–Kutta method. The result is shown in Figure 2, where the measurand, $x\left(t\right)$ is compared with uncompensated result, $y\left(t\right)/k_{0}$, where $y\left(t\right)$ is the instrument indication (voltage). Therefore, the signals in the figure are both accelerations and can thus be directly compared. The amplification of the amplitude and the phase delay effect can be noted.

Then the discretized version of the continuous system (18) was considered, assuming a sampling interval ∆t=0.2 ms, and adopting the zero-hold approximation method [26]. The following values were obtained for the parameters of the discrete difference Equation (8):

- $a_{1}=0.4951$,
- $a_{2}=-0.2846$,
- $b_{1}=0.0240$, and
- $b_{2}=0.0155$.

**Figure 3:**
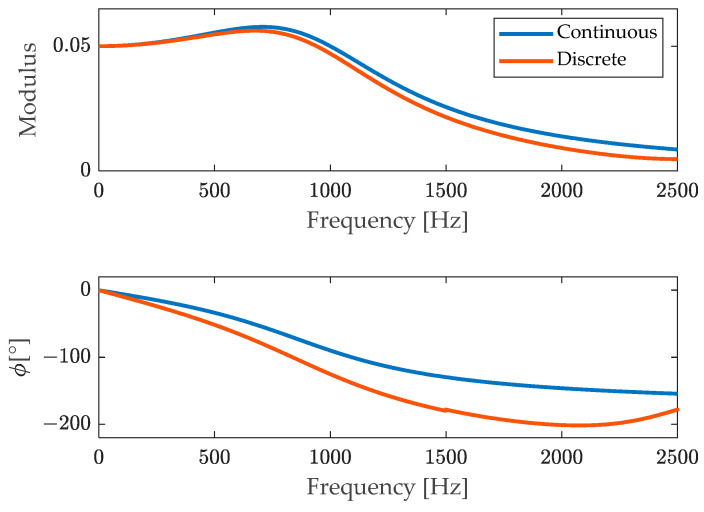
Comparison of the frequency response of the original system and of the corresponding one coming from the discrete model.

In order to check that the finite difference equation provides a good approximation of the original continuous-time differential Equation (18), the corresponding frequency responses were compared, as shown in Figure 3.

**Figure 4:**
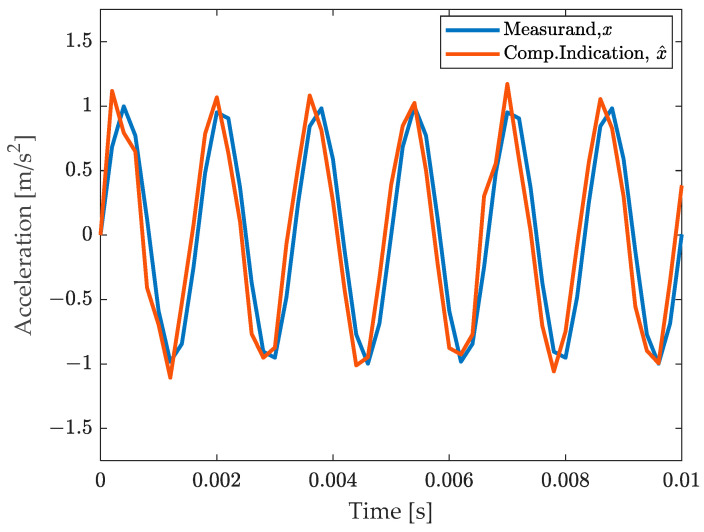
Comparison of the measurand with the final, compensated measurement result.

The effectiveness of the procedure can be appreciated by comparing the compensated measurement result with the measurand in Figure 4. It clearly appears that both the amplitude and the phase effects have been visibly compensated. The phase appears slightly anticipated, yet it should be noted that the output of the filter is produced with a delay of ∆t, and thus this anticipation is a postprocessing effect.

**Figure 5:**
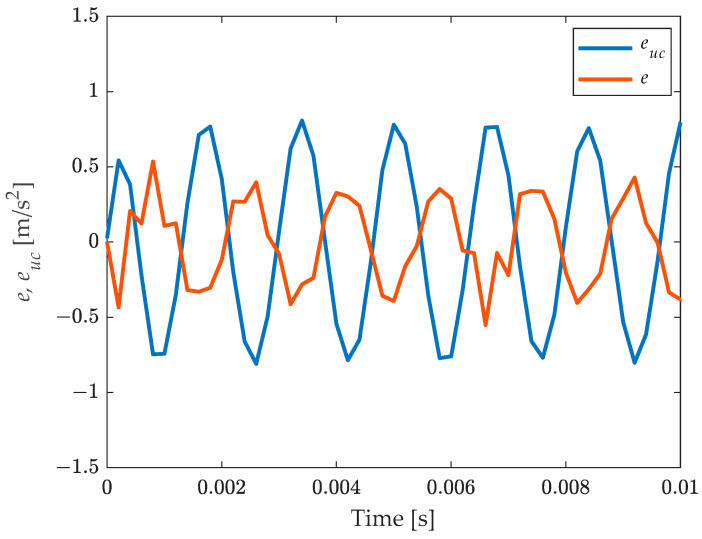
Comparison of the error with uncompensated and compensated result.

Figure 5 presents the error for compensated output

(20) $$e\left(t\right)=\hat{x}\left(t\right)-x\left(t\right),$$

together with the error for the un-compensated output:

(21) $$e_{uc}\left(t\right)=k_{0}^{-1}y\left(t\right)-x\left(t\right).$$

The benefit of compensation is evident. The phase shift is explained considering the comment to Figure 4.

### 3.3. Simulated Step-like Phenomenon

**Figure 6:**
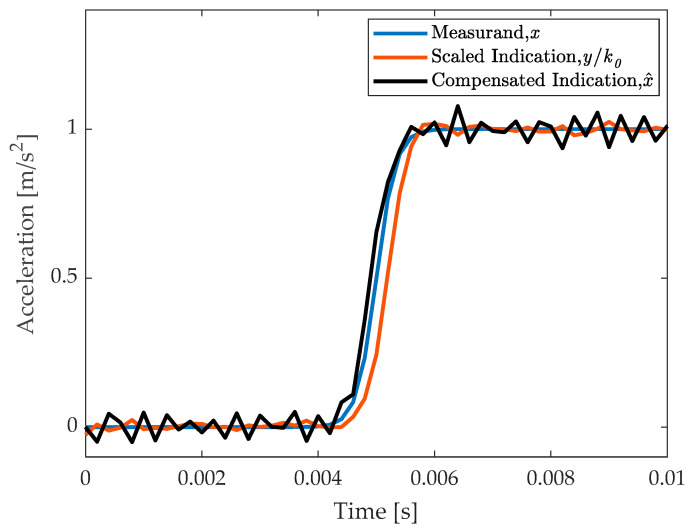
Dynamic compensation for a step-like signal: the measurand is compared with the compensated and with the uncompensated results.

As an additional example, a step-like signal, with additional noise, was considered. The signal was simulated by a sigmoidal function:

(22) $$x\left(t\right)=x_{0}\frac{1}{1+e^{-\frac{\left(t-t_{0}\right)}{T}}}\;,$$

with $x_{0}=1.0\;\frac{m}{s^{2}}$, $t_{0}=5\;\mathrm{ms}$, and T=0.167 ms. For observation noise, an rms value of 0.1 mV was assumed. Results are shown in Figure 6.

It appears that dynamic compensation allows us to eliminate dynamic effects but with some increase in noise. Therefore, this method is particularly convenient when dynamic effects are considered more critical than noise.

To sum up, simulation requires the following steps:

- Definition of the differential equation describing the dynamic behaviour of the measuring instrument, Equations (17) and (18), with its parameters;
- Discretization of the continuous time equation (for example with zero hold method) to obtain a discrete time Equation (8);
- Definition of the II order linear compensation filter from the discrete time equation coefficients (14);
- Use the system for a dynamical measurement of a signal $x\left(t\right),$ with proper sampling frequency, to obtain an array of readings $y_{i}$;
- Application of the compensation filter (14) to the readings to obtain the compensated measurement result ${\hat{x}}_{i}$.

In the experimental validation we will follow the same steps.

## 4. Experimental Validation of the Method

The method validation was carried out in two steps. At first, the frequency response of the accelerometer to be tested was obtained for the given mounting condition. Then, the accelerometer was subjected to a set of inputs, measured also by a reference device, and the performance of the accelerometer under test was evaluated both with and without compensation.

Tested inputs include:

1. Two mono-harmonic signals: a signal with one sinusoidal component with a frequency within the bandwidth of the accelerometer under test and the other having a frequency out of band and near the accelerometer resonant frequency (see Section 4.1);
2. A bi-harmonic signal: a signal with two sinusoidal components, one with a frequency in band and the other with a frequency out of band (Section 4.2);
3. A multicomponent signal including several in-band and out-of-band harmonic components (see Section 4.3).

**Figure 7:**
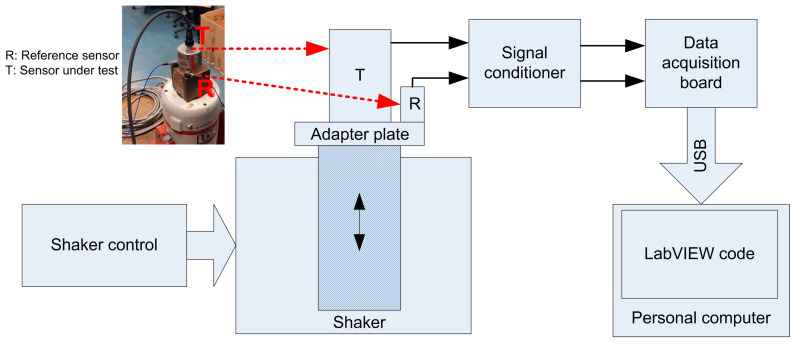
The experimental setup.

The experimental set up consisted in two accelerometers excited by an electromagnetic shaker and mounted on a shaker’s adapter plate. The plate and fixation methods for the accelerometers (screw for the accelerometer under test and epoxy for the reference) can be considered rigid in the frequency band considered [27,28]. In the test setup, the sensor to be tested (T) was a low frequency accelerometer (PCB model 393B31), whilst an accelerometer with a higher bandwidth compared to T (PCB model 333B30) acted as the reference (R) (see Figure 7). Some important technical details of the sensors involved in the experiment are reported in Table 1.

The experimental setup is shown in Figure 7.

**Figure 8:**
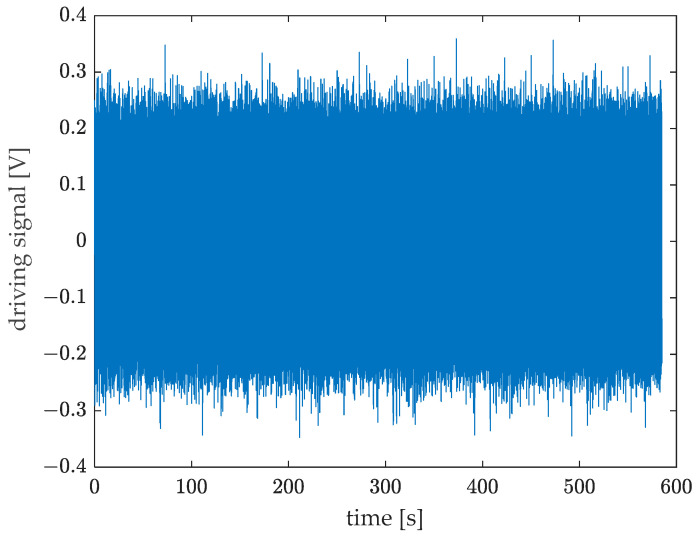
Shaker driving signal for random excitation.

**Figure 9:**
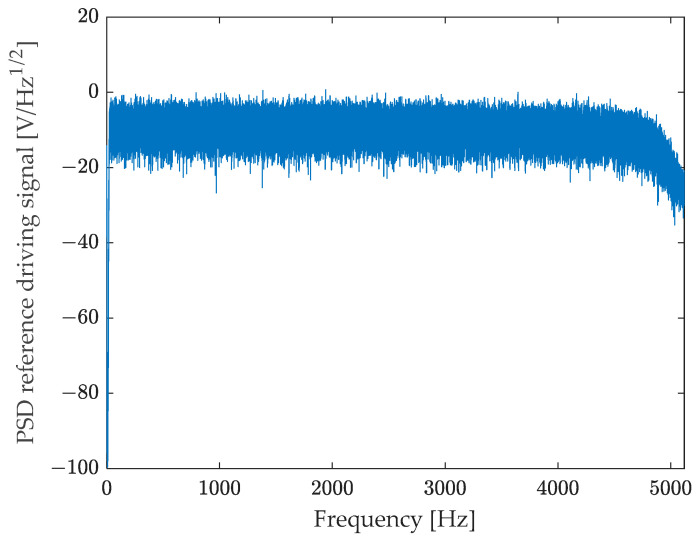
Power spectral density of the driving signal.

Vibrations were generated by means of an electromagnetic shaker driven with a random signal (see Figure 8) in the 20–4500 Hz frequency band (see Figure 9). The signal was sampled with a sampling rate $f_{s}=10,240\;\mathrm{Hz}$ and acquired for about 600 s.

**Figure 10:**
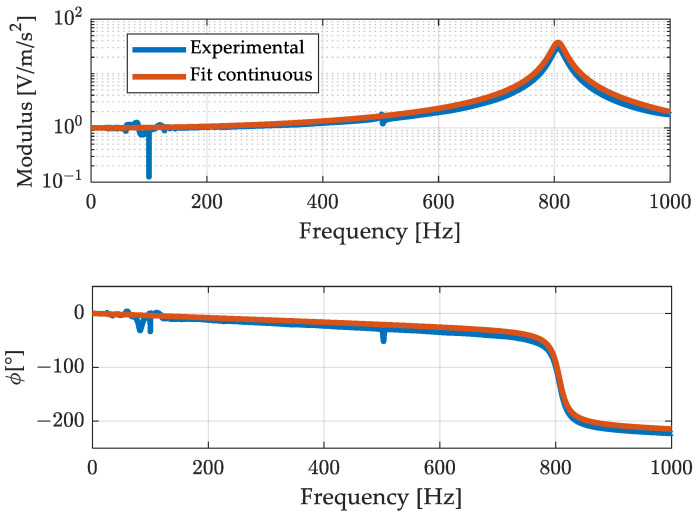
Experimental frequency response function and its fit with a two-poles, two-zeros continuous time model.

The frequency response $a_{T}\left(f\right)/a_{R}\left(f\right)$, where $a_{T}$ and $a_{R}$ are the acceleration measured by the tested and reference accelerometer, respectively, was then obtained using the non-parametric Welch method and the H1 estimator for the frequency response function [29,30]. The estimate was obtained using time windows of 10 s each and an overlap of 66%. Then, the experimental frequency response function, represented in Figure 10, was fitted with a model with two poles and two zeros to take into account the dynamics of both the accelerometers (see Figure 7).

The poles of the fitting model were extracted and the continuous time model of the tested accelerometer was derived as:

(23) $$H_{y/a}\left(\omega \right)=\frac{k_{0}\omega_{0}^{2}}{-\omega^{2}+2j\zeta_{o}\omega_{0}\omega +\omega_{0}^{2}},$$

with $k_{0}=0.985\;\frac{\mathrm{mV}}{m/s^{2}}$, $\omega_{0}=806.53\cdot 2\pi \frac{\mathrm{rad}}{s}$ and $\zeta_{0}=0.014$.

Then, the identified continuous time system (23) was discretized by the z-hold method, with ∆t=19.531 μs. This sampling interval was chosen to meet that of the signals used for testing the method (see Section 4.1, Section 4.2 and Section 4.3). The obtained discrete time equation was:

(24) $$y_{i}=1.083y_{i-1}-0.9727y_{i-2}+0.4403x_{i-1}+0.4361x_{i-2}+w_{i}\;,$$

where $y_{i}$ is the (discretized) output voltage $\left(V\right)$ and $x_{i}$ is the input acceleration $\left(m/s^{2}\right)$.

**Figure 11:**
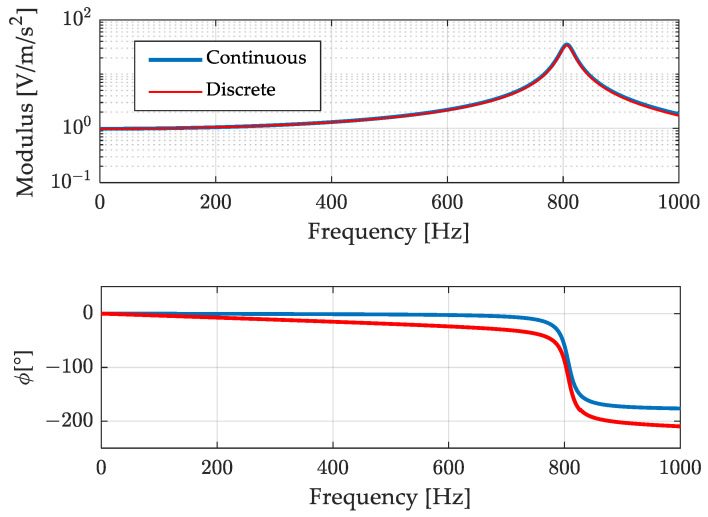
Comparison of the continuous frequency response function with its discrete version.

In Figure 11, the continuous model of the frequency response (23) is compared to the discrete one corresponding to the finite difference Equation (24).

Looking at Figure 11 it can be noticed that the approximation of the modulus is very good, whilst the phase departs somewhat to the original.

### 4.1. Test Case 1: Pure Sine (in Band and out of Band)

In this first test case, the compensation was applied to two different sinusoidal signals: one out of band at 700 Hz and another in band at 160 Hz.

It is worth remembering that the accelerometer under test considered has a nominal resonant frequency > 700 Hz and an experimentally estimated resonant frequency of about 800 Hz (see Figure 10); therefore, the sinusoidal component at 700 Hz is significantly out of the accelerometer bandwidth (i.e., 200 Hz, see Table 1) and near the resonance for the device under test.

**Figure 12:**
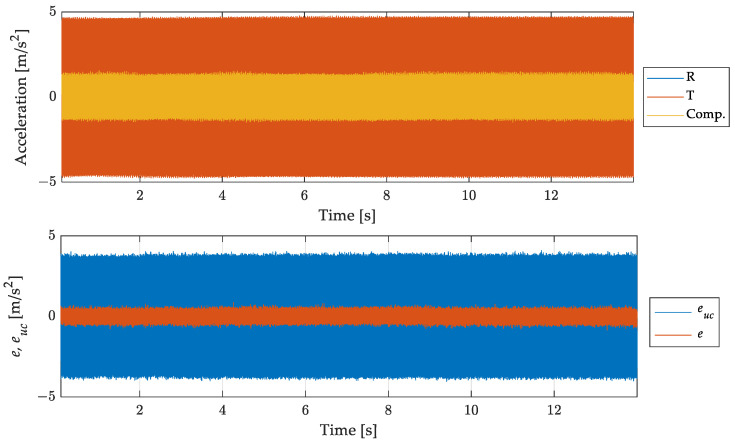
Comparison of the measurand with the (uncompensated) scaled instrument indication and the compensated measurement result. Full acquisition time histories (**top**) and errors for uncompensated $e_{uc}$ and compensated result *e* (**bottom**).

**Figure 13:**
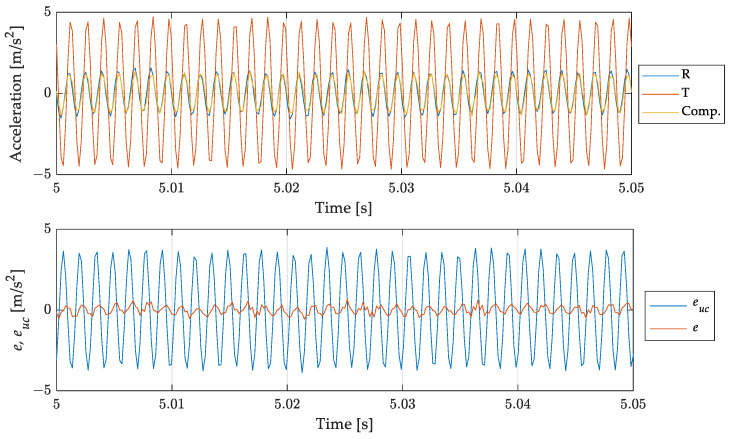
Sinusoidal signal at 700 Hz: comparison of the measurand with the (uncompensated) scaled instrument indication and the compensated measurement result. Short time histories (**top**) and errors for un-compensated $e_{uc}$ and compensated result *e* (**bottom**).

Results related to the 700 Hz out-of-band signal are shown in Figure 12 and Figure 13. Figure 12 shows, in the top graph, the overall acquisition of the signals measured by the reference accelerometer, by the one under test, and the signal of the accelerometer under test after the compensation process. From this figure it is possible to notice that the filtering process aimed at compensating the signal does not lead to instabilities, or long transient effects. Figure 13 is a zoom of Figure 12 where signals are presented in a duration of few hundredths of a second. The reference (measurand), the un-compensated (under test), and the compensated (SDOF filtered) results are compared in the upper graph. The improvement in modulus is remarkable. Concerning the phase some anticipation can be noted, in contrast with the delay of the uncompensated result. This effect is due to the phase difference between the discrete and the continuous models as shown in Figure 11. It can be noted that such anticipation is of the order of one sampling interval and can be reduced if the sampling frequency is increased. However, this oversampling can lead to an increase of the noise in the compensated measurement and, therefore, it can be used when the signal-to-noise ratio is high.

The bottom graph of Figure 12 and Figure 13 shows the difference between the reference and the uncompensated and compensated signals, evidencing the effectiveness of the proposed compensation method in reducing the error due to the use of the accelerometer for measuring a signal out of its bandwidth.

**Figure 14:**
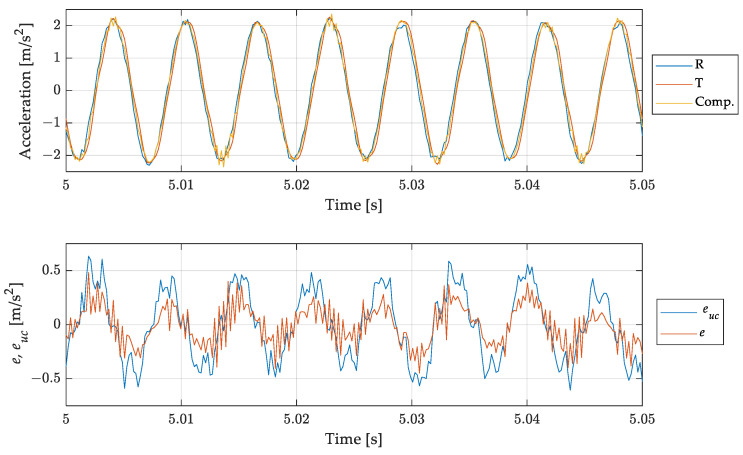
Test case 1, sinusoidal signal at 160 Hz: comparison of the measurand with the (uncompensated) scaled instrument indication and the compensated measurement result. Short time histories (**top**) and errors for uncompensated $e_{uc}$ and compensated result *e* (**bottom**).

To verify the reliability of the procedure also when signals inside the bandwidth of the sensor are measured, a sinusoidal input at 160 Hz has been considered. Figure 14 shows results similar to those shown in Figure 13: the compensation procedure does not compromise the measurement result. The compensated and uncompensated signals are in good agreement, and the anticipation introduced by the filter is smaller than that observed in the previous case due to the higher accuracy of the estimated frequency response function in the considered frequency band with respect to the previous case. However, it is worth underlining that the filtering procedure leads, as mentioned, to a decrease in the signal-to-noise ratio (see Figure 14, bottom).

### 4.2. Test Case 2: Two Components Signal (out of Band + in Band)

**Figure 15:**
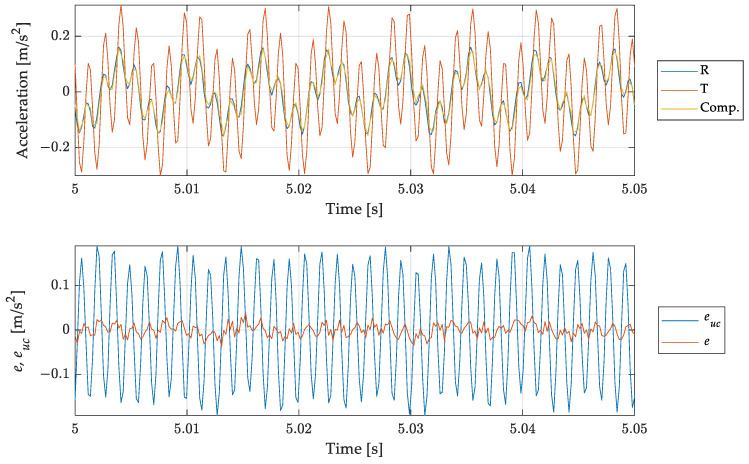
Test case 2, periodic signal with two harmonic components at 160 Hz and 700 Hz: comparison of the measurand with the (uncompensated) scaled instrument indication and the compensated measurement result. Short time histories (**top**) and errors for uncompensated $e_{uc}$ and compensated result *e* (**bottom**).

In this test case, the compensation filter was applied to a harmonic signal with two components of equal amplitude: an in-band component at 160 Hz (close to the upper limit of the device under test, which is 200 Hz, see Table 1) and an out-of-band component at 700 Hz. Results are presented in Figure 15 in terms of time histories of the reference, measured and compensated signals together with the dynamic measurement error.

Even in this case the compensation filter operates a clear reduction of the dynamic measurement error, without compromising the contribution of the in-band signal component. This test case allows demonstrating the reliability of the proposed approach even when a part of the signal is within the bandwidth of the considered device, so it would not require a compensation.

### 4.3. Test Case 3: Multicomponent

Lastly, the compensation filter was applied to a complex pluri-harmonic signal composed by 10 sinusoidal components both in band and out of band whose frequency content is reported in Table 2. Particularly, this test case considers a signal having four in band, one at the limit, and six out-of-band components, going up to a frequency near the experimental resonant frequency as in test case 1. Amplitudes were chosen equal for all the components.

**Figure 16:**
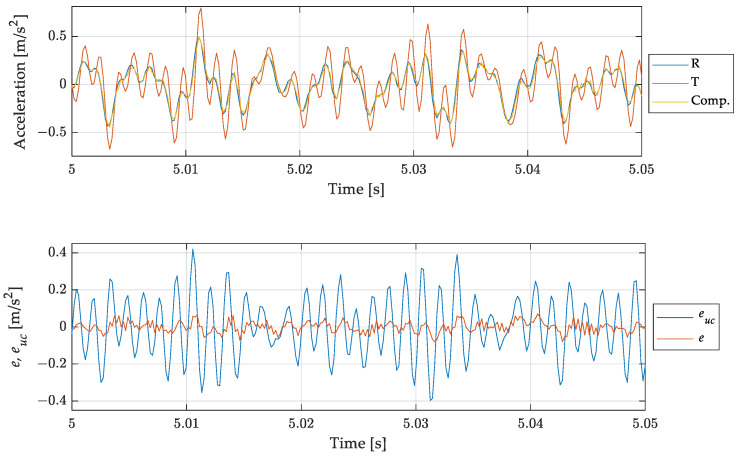
Test case 3: multicomponent signal. Comparison of the measurand with the (uncompensated) scaled instrument indication and the compensated measurement result. Short time histories (**top**) and errors for uncompensated $e_{uc}$ and compensated result *e* (**bottom**).

Results are presented in Figure 16 in terms of time histories of the signals and the errors as in the previous cases.

Test case 3 was chosen to test the filter behaviour in a demanding case, including several components to be compensated, each one in a different way, and several components to be kept unaltered.

As shown in Figure 16, the proposed approach proved to be effective in this case also, leading to a drastic reduction of the dynamic error.

### 4.4. Result Discussion

The three test cases proposed—Figure 13, Figure 15 and Figure 16—were chosen to increasingly stress the working conditions of the accelerometer under test, obtaining always a significant reduction, about ten times, in the dynamic error for the compensated output. However, as shown by the error trend in Figure 13, Figure 14, Figure 15 and Figure 16, the compensation does not lead to a null error, as expected, but this error shows a deterministic component which cannot be ascribed to the different noise level of the considered sensors and to the additional noise brought by the filtering process. The deterministic part of the difference between the reference and the compensated signal depends on the accuracy of the filter in describing the behaviour of the tested sensor. Indeed, looking closely Figure 10 and Figure 11, it is possible to notice that there are differences between the experimental and the estimated frequency response function of the sensor, both in terms of phase and amplitude which imply a residual difference between the compensated signal and the reference. To verify the consistency between the error obtained in the different test cases and the theoretical one ascribed to the inaccuracy of the filter used, it is possible to make a comparison in terms of the ratio of the mean square value of the compensated $\Psi_{ec}^{2}$, and the uncompensated error $\Psi_{euc}^{2}$:

(25) $$r_{exp}=\frac{\Psi_{ec}^{2}}{\Psi_{euc}^{2}},$$

where the mean square value of the compensated and uncompensated error can be obtained as:

(26) $$\Psi_{e}^{2}=\mu^{2}+\sigma^{2},$$

where μ is the mean value of the time history of the considered signal and σ its standard deviation.

As for the expected mean square value of the error signals, harmonic components of equal amplitude A, different frequencies $\omega_{i}$, and phases $\varphi_{i}$ are considered. The mean square values of the uncompensated and compensated error can be estimated respectively as:

(27) $$\Psi_{euc}^{2}=nA^{2}\sum_{i=1}^{n}\left[\frac{1}{2}+\frac{H_{i,th}^{2}\left(\omega_{i}\right)}{2}-H_{i,th}\left(\omega_{i}\right)\cos\left(\varphi_{i,th}\left(\omega_{i}\right)\right)\right],\;\Psi_{ec}^{2}=nA^{2}\sum_{i=1}^{n}\left[\frac{1}{2}+\frac{H_{i,th}^{2}\left(\omega_{i}\right)}{2{\hat{H}}_{i}^{2}\left(\omega_{i}\right)}-\frac{H_{i,th}\left(\omega_{i}\right)}{{\hat{H}}_{i}\left(\omega_{i}\right)}\cos\left(\varphi_{i,th}\left(\omega_{i}\right)-{\hat{\varphi }}_{i}\left(\omega_{i}\right)\right)\right],$$

where i indicates the *i-th* harmonic component of the signal, the subscript −th is related to the amplitude and phase of the frequency response function of the tested sensor evaluated experimentally (see Figure 10), and the ^ refers instead to amplitude and phases of the estimated discrete frequency response (see Figure 11).

Substituting Equation (27) in (25), it is possible to obtain the expected error reduction:

(28) $$r_{th}=\frac{\sum_{i=1}^{n}\left[\frac{1}{2}+\frac{H_{i,th}^{2}\left(\omega_{i}\right)}{2{\hat{H}}_{i}^{2}\left(\omega_{i}\right)}-\frac{H_{i,th}\left(\omega_{i}\right)}{{\hat{H}}_{i}\left(\omega_{i}\right)}\cos\left(\varphi_{i,th}\left(\omega_{i}\right)-{\hat{\varphi }}_{i}\left(\omega_{i}\right)\right)\right]}{\sum_{i=1}^{n}\left[\frac{1}{2}+\frac{H_{i,th}^{2}\left(\omega_{i}\right)}{2}-H_{i,th}\left(\omega_{i}\right)\cos\left(\varphi_{i,th}\left(\omega_{i}\right)\right)\right]}.$$

The results in terms of $r_{th}$ and $r_{exp}$ are shown in Table 3 for all the test-cases considered. Comparing the results, it is possible to notice that the filtering process provides an error attenuation which is in agreement with the theoretical expectation given the estimated and measured sensor frequency response. The difference between the two values can be ascribed to the uncertainties associated to the estimates of the amplitude and the phases used in (27), and the different noise level associated with the reference and tested accelerometers. However, Test case 1b, associated with the in-band mono-harmonic signal, deserves a deeper analysis. Looking at the $r_{th}$ value, it is possible to notice that it does not assume a value higher than 100%, as expected (i.e., the compensation should introduce an error due to the differences between the estimated and experimental frequency response of the sensor tested), but the compensation reduces the error, which is about 20% of the uncompensated one. This behaviour can be explained by the compensation, caused by the filter, of the small delay between the reference and measured signal—see Figure 14. Moreover, it is worth noticing that both $r_{th}$ and $r_{exp}$ assume higher values with respect to the other test cases. This is due to the small mean square value of the uncompensated error $\Psi_{euc}^{2}$ when considering an in band signal—see (25). Finally, the difference between the estimated and the theoretical error reduction is higher with respect to the other cases. This effect is a consequence of the filtering process, which leads, in this case, to an increase of the noise on the compensated signal—see Figure 14.

## 5. Conclusions

A method for the dynamic compensation of accelerometers that is the outcome of a general probabilistic model of the measurement process was presented, illustrated, and discussed. The proposed method up to now had been fully developed and tested only for first-order measurement systems. Here, it is applied to an accelerometer described by a second-order model. This constitutes a major step in the research program of the authors, which ultimately aims at dealing n-th order (causal) systems since the transition from a first to a second order involves moving from scalar to vector systems, which is a key point. Furthermore, second-order systems are particularly significant since they may constitute local approximations nearby the resonance frequency even of systems of higher order. This is in fact the case of piezoelectric accelerometers, which also exhibit a low frequency high-pass behaviour due to the piezoelectric transducer.

The method has been carefully validated by simulation and experimentation. Such a validation, in particular, confirmed that the hypothesis assumed for the implementation of the method, i.e., that at the i-th step, the value of the measurand, can be replaced by its estimate obtained at the previous step—see comment just before (14)—and the use of a second-order model approximating the behaviour of the system nearby the resonance frequency were appropriate. We suggest that this confirms the applicability of the method in other similar important cases such as the case of piezoelectric force transducers.

Therefore, to sum up, a method for the dynamic compensation of a second-order device (i.e., an accelerometer) was developed and tested. The method directly comes from a general probabilistic model of the measurement system, whose basic structure is virtually common to all types of measurement. This may be of interest in a theoretical and epistemological perspective for putting dynamic measurement on a sound foundation rather than simply taking methods and results from system and signal theory. The validation of the method for accelerometers may encourage its application for this type of sensors and other similar, such as, e.g., force transducers.

## Figures and Tables

**Table 1:** Reference (R) and under test (T) accelerometers technical specifications (nominal values).

	PCB 333B30 (R)	PCB 393B31 (T)
Mass (kg)	0.004	0.635
Sensitivity (mV/ms^−2^)	10.2	1020
±5% bandwidth (Hz)	0.5–3000	0.1–200
Resonant frequency (kHz)	≥40	≥0.7

**Table 2:** Test case 3. Frequencies for the multi-harmonic signal. Refer to Figure 8 and Table 1 for device under test data.

**Multicomponent frequencies (Hz)**		160	170	180	200	300	350	400	600	650	700	
**Accelerometer under test bandwidth and resonant frequency (Hz)**	0.07				200							806

**Table 3:** RMS values for dynamic errors in the three test cases.

Test Case	$r_{th}$ (Equation (28)) %	$r_{exp}$ (Equation (25)) %
1a: mono-harmonic, out of band	0.76	0.93
1b: mono-harmonic, in band	20.30	35.14
2: bi-harmonic	0.85	1.42
3: multi-harmonic	1.86	3.60

## Data Availability

The data presented in this study are available on request from the corresponding author.
